# Dr. Madison H. Rose

**Published:** 1905-02

**Authors:** 


Dr. Madison H. Rose, of Thornton, Ind., a graduate of the University of Buffalo in 1861, died at his home, December 16, 1904, aged 72 years. Dr. Rose served as a medical officer during the civil war, attaining the rank of surgeon.

